# Application of Impedance Microbiology for Evaluating Potential Acidifying Performances of Starter Lactic Acid Bacteria to Employ in Milk Transformation

**DOI:** 10.3389/fmicb.2016.01628

**Published:** 2016-10-17

**Authors:** Elena Bancalari, Valentina Bernini, Benedetta Bottari, Erasmo Neviani, Monica Gatti

**Affiliations:** ^1^Laboratory of Food Microbiology, Department of Food Science, University of ParmaParma, Italy; ^2^Multidisciplinary Interdepartmental Dairy Cente, University of ParmaParma, Italy

**Keywords:** impedance micorbiology, lactic acid bacteria, starter activity, BacTrac, Gompertz model

## Abstract

Impedance microbiology is a method that enables tracing microbial growth by measuring the change in the electrical conductivity. Different systems, able to perform this measurement, are available in commerce and are commonly used for food control analysis by mean of measuring a point of the impedance curve, defined “time of detection.” With this work we wanted to find an objective way to interpret the metabolic significance of impedance curves and propose it as a valid approach to evaluate the potential acidifying performances of starter lactic acid bacteria to be employed in milk transformation. To do this it was firstly investigated the possibility to use the Gompertz equation to describe the data coming from the impedance curve obtained by mean of BacTrac 4300®. Lag time (λ), maximum specific M% rate (μmax), and maximum value of M% (Yend) have been calculated and, given the similarity of the impedance fitted curve to the bacterial growth curve, their meaning has been interpreted. Potential acidifying performances of eighty strains belonging to *Lactobacillus helveticus, Lactobacillus delbrueckii* subsp. *bulgaricus, Lactococcus lactis*, and *Streptococcus thermophilus* species have been evaluated by using the kinetics parameters, obtained from Excel add-in DMFit version 2.1. The novelty and importance of our findings, obtained by means of BacTrac 4300®, is that they can also be applied to data obtained from other devices. Moreover, the meaning of λ, μmax, and Yend that we have extrapolated from Modified Gompertz equation and discussed for lactic acid bacteria in milk, can be exploited also to other food environment or other bacteria, assuming that they can give a curve and that curve is properly fitted with Gompertz equation.

## Introduction

Impedance microbiology is a rapid method that enables qualitative and quantitative tracing of microorganisms by measuring the change in the electrical conductivity. It is based on a principle that dates back to 1899 (Stewart, 1899) but its application to food microbiology field is most recent and mainly associated to rapid detection of foodborne pathogenic bacteria (Yang and Bashir, 2007).

Impedance, applied to microbiology, can be defined as the resistance to flow of an alternating current that passes through a conducting microbial growth medium. During microbial growth, metabolic processes produce electrically measurable changes in the growth medium due to the metabolism of high-molecular weight nutrients into smaller charged ionic components that increase the electrical conductivity of the medium. Variation in electrical conductivity, monitored during time, is proportional to the change in the number of microorganisms and therefore the microbial growth can be measured (Batrinou et al., 2005).

Different systems, able to perform this measurement, are available. In the past, the most common commercial equipments used for impedance microbiology were, RABIT system™ (Don Whitley Scientific, Shipley, UK), Bactometer™ (bioMerieux, Marci l'Etolie, France) and Malthus™ (Malthus Instrument, Crawley, England). A more recent equipment is the BacTrac™ microorganism growth analyser (SyLab, Purkersdorf-Vienna, Austria). Common to all the systems is the measurement of an electronic signal that quantify the movement of ions between two electrodes (conductance) while, in some devices, the storage of charge at the electrodes medium interface (capacitance) is also measured (Noble et al., 1999). Plotting of the continuous measurement of cumulative increase in conductance, or capacitance, graphically results in an impedance curve (Rediers et al., 2012). The most common way to use this curve in microbiological analysis is fixing a point, generally defined as “time of detection.” However, fixed the microorganism, medium and temperature, this point varies between one device and another. Time to detection (TTD) for RABIT corresponds to the point where the cumulative change in conductivity from the baseline meets or exceeds a set value over a defined time interval (Rediers et al., 2012). Detection Time (DT) for Malthus is obtained when a change in conductance over a threshold reference value set by the operator is observed (Lanzanova et al., 1993). DT of Bactometer is the amount of time required to cause a series of significant deviations from baseline impedance values (Noble et al., 1999). DT for Backtrack is the time when the impedance curve meets the threshold level of 5% (Čurda and Plocková, 1995). Indeed, the “time of detection” is the principal parameter measured by all the devices and it coincides with the reaching of a cells concentration of about 10^6^–10^7^ cells per ml (Noble et al., 1999) thus, it is strongly affected by bacterial cells physiological state (Lanzanova et al., 1993). this parameter is largely used to monitoring pathogens or spoiling bacteria in food (Gracias and McKillip, 2004), and also antimicrobial activity (Marino et al., 2001; Silva et al., 2003; Kunicka-Styczyńska and Gibka, 2010) included lytic infections by bacteriophages (Amorim et al., 2009). Recently, an intriguing unconventional approach to impedance microbiology was considered to detect bacteriophages responsible for cell lysis (Mortari et al., 2015). However, to the author's knowledge, the significance of the whole impedance curve have never been objectively related to microbial behavior.

The responses of microorganisms to specific environmental conditions, such as temperature, pH and a_w_, can be described by predictive microbiology, a sub-discipline of food microbiology dealing with the development of mathematical models (Baranyi and Roberts, 1995). Several models have been developed to represent and predict microbial growth or inactivation in food and, nowadays, such models can be very useful in food technology and processing since they are applied to predict the outcome of fermentation processes under particular circumstances and to assess the effects of environmental conditions on microbial growth. Examples of primary models, widely applied to describe the growth of lactic acid bacteria, include sigmoidal equations, such as Logistic and Modified Gompertz models (Chowdhury et al., 2007; Slongo et al., 2009). This describes the changes of the microbial population density as a function of time using a limited number of kinetic parameters (e.g., lag time, growth or inactivation rate and maximum population density) while it is not taken into account the stage of death. The Gompertz model provides a convenient mathematical tool that approximates the way in which microbiologists have traditionally estimated the graph of the growth kinetics (Buchanan et al., 1997).

Aim of this work was firstly to investigate the possibility to use the Gompertz equation to describe the data coming from the impedance curve obtained by mean of BacTrac 4300® and, secondly, to use the so described kinetics parameters, to evaluate the potential acidifying performances of several lactic acid bacteria strains for their possible use as starters in milk transformation.

## Materials and methods

### Strains, media, and growth conditions

Eighty strains representing four starter lactic acid bacteria species, *Lactobacillus helveticus, Lactobacillus delbrueckii* subsp. *bulgaricus, Lactococcus lactis*, and *Streptococcus thermophilus* (Table 1), were analyzed by impedance measurements. The strains, belonging to the collection of the Laboratory of Food Microbiology of the Department of Food Science of University of Parma, have been previously isolated from dairy matrixes and identified by16S rRNA sequencing.

**Table 1:** **Lactic acid bacteria strains used in this study**.

**Species**	**Strain**	**Source**	**Origin**
*Lactobacillus delbrueckii* subsp. *bulgaricus*	260	Clerici-Sacco Group	Commercial starter
*Lactobacillus delbrueckii* subsp. *bulgaricus*	265	UNIPR	Natural whey starter
*Lactobacillus delbrueckii* subsp. *bulgaricus*	308	UNIPR	Natural whey starter
*Lactobacillus delbrueckii* subsp. *bulgaricus*	1865	UNIPR	Natural whey starter
*Lactobacillus delbrueckii* subsp. *bulgaricus*	1932	UNIPR	Curd
*Lactobacillus delbrueckii* subsp. *bulgaricus*	1982	UNIPR	Curd
*Lactobacillus delbrueckii* subsp. *bulgaricus*	2000	UNIPR	Curd
*Lactobacillus delbrueckii* subsp. *bulgaricus*	2225	UNIPR	Milk
*Lactobacillus delbrueckii* subsp. *bulgaricus*	2230	UNIPR	Milk
*Lactobacillus delbrueckii* subsp. *bulgaricus*	3436	UNIPR	Cheese
*Lactobacillus delbrueckii* subsp. *bulgaricus*	4622	UNIPR	Italian yogurt
*Lactobacillus delbrueckii* subsp. *bulgaricus*	4623	UNIPR	Italian yogurt
*Lactobacillus delbrueckii* subsp. *bulgaricus*	4624	UNIPR	Italian yogurt
*Lactobacillus delbrueckii* subsp. *bulgaricus*	4625	UNIPR	Italian yogurt
*Lactobacillus delbrueckii* subsp. *bulgaricus*	4626	UNIPR	Italian yogurt
*Lactobacillus delbrueckii* subsp. *bulgaricus*	4627	UNIPR	Italian yogurt
*Lactobacillus delbrueckii* subsp. *bulgaricus*	4628	UNIPR	Italian yogurt
*Lactobacillus delbrueckii* subsp. *bulgaricus*	4629	UNIPR	Italian yogurt
*Lactobacillus delbrueckii* subsp. *bulgaricus*	LMG 6901	NCIMB	Bulgarian yogurt
*Lactobacillus delbrueckii* subsp. *bulgaricus*	LMG 12168	NCIMB	Homemade yogurt
*Lactobacillus helveticus*	1	UNIPR	Natural whey starter
*Lactobacillus helveticus*	2	UNIPR	Natural whey starter
*Lactobacillus helveticus*	3	UNIPR	Natural whey starter
*Lactobacillus helveticus*	4	UNIPR	Natural whey starter
*Lactobacillus helveticus*	5	UNIPR	Natural whey starter
*Lactobacillus helveticus*	6	UNIPR	Natural whey starter
*Lactobacillus helveticus*	8	UNIPR	Natural whey starter
*Lactobacillus helveticus*	9	UNIPR	Natural whey starter
*Lactobacillus helveticus*	11	UNIPR	Natural whey starter
*Lactobacillus helveticus*	13	Clerici-Sacco Group	Commercial starter
*Lactobacillus helveticus*	23	UNIPR	Natural whey starter
*Lactobacillus helveticus*	28	UNIPR	Natural whey starter
*Lactobacillus helveticus*	35	UNIPR	Natural whey starter
*Lactobacillus helveticus*	36	UNIPR	Natural whey starter
*Lactobacillus helveticus*	37	UNIPR	Natural whey starter
*Lactobacillus helveticus*	39	UNIPR	Natural whey starter
*Lactobacillus helveticus*	41	UNIPR	Natural whey starter
*Lactobacillus helveticus*	42	UNIPR	Natural whey starter
*Lactobacillus helveticus*	1697	UNIPR	Natural whey
*Lactobacillus helveticus*	2457	UNIPR	Cheese
*Lactoccoccus lactis*	220	Clerici-Sacco Group	Commercial starter
*Lactoccoccus lactis*	225	Clerici-Sacco Group	Commercial starter
*Lactoccoccus lactis*	662	UNIPR	Milk
*Lactoccoccus lactis*	663	UNIPR	Milk
*Lactoccoccus lactis*	664	UNIPR	Milk
*Lactoccoccus lactis*	667	UNIPR	Milk
*Lactoccoccus lactis*	672	UNIPR	Milk
*Lactoccoccus lactis*	674	UNIPR	Milk
*Lactoccoccus lactis*	1239	UNIPR	Milk
*Lactoccoccus lactis*	1426	UNIPR	Milk
*Lactoccoccus lactis*	1428	UNIPR	Milk
*Lactoccoccus lactis*	1439	UNIPR	Milk
*Lactoccoccus lactis*	2269	UNIPR	Milk
*Lactoccoccus lactis*	2270	UNIPR	Milk
*Lactoccoccus lactis*	2271	UNIPR	Milk
*Lactoccoccus lactis*	4062	UNIPR	Cheese
*Lactoccoccus lactis*	4064	UNIPR	Cheese
*Lactoccoccus lactis*	4065	UNIPR	Cheese
*Lactoccoccus lactis*	4067	UNIPR	Cheese
*Lactoccoccus lactis*	4068	UNIPR	Cheese
*Streptococcus thermophilus*	83	UNIPR	Milk
*Streptococcus thermophilus*	84	UNIPR	Milk
*Streptococcus thermophilus*	95	UNIPR	Curd
*Streptococcus thermophilus*	111	UNIPR	Milk
*Streptococcus thermophilus*	113	UNIPR	Curd
*Streptococcus thermophilus*	114	UNIPR	Curd
*Streptococcus thermophilus*	140	UNIPR	Curd
*Streptococcus thermophilus*	145	UNIPR	Milk
*Streptococcus thermophilus*	159	UNIPR	Milk
*Streptococcus thermophilus*	160	UNIPR	Milk
*Streptococcus thermophilus*	161	UNIPR	Milk
*Streptococcus thermophilus*	162	UNIPR	Milk
*Streptococcus thermophilus*	163	UNIPR	Milk
*Streptococcus thermophilus*	176	UNIPR	Curd
*Streptococcus thermophilus*	192	UNIPR	Milk
*Streptococcus thermophilus*	410	UNIPR	Milk
*Streptococcus thermophilus*	526	UNIPR	Milk
*Streptococcus thermophilus*	530	UNIPR	Curd
*Streptococcus thermophilus*	547	UNIPR	Curd
*Streptococcus thermophilus*	4028	UNIPR	Curd

Strains, maintained as frozen stocks cultures in MRS (Oxoid, Ltd., Basingstoke, United Kingdom) (*Lactobacillus*), or M17 (Oxoid Ltd.) (*Lactococcus* and *Streptococcus*) broth containing 20% (v/v) glycerol at −80°C, were recovered in MRS or M17 broth by two overnight sub-culturing (5% v/v) at 42°C for *Lactobacillus* and *Streptococcus*, and 30°C for *Lc. lactis*. Then, other 28 h sub-culturing (5% v/v) of each strain in skim milk powder (Oxoid Ltd.), reconstituted to 10% (w/v) and sterilized at 110°C for 30 min (SSM), were performed before use.

### Impedance measurement

A BacTrac 4300® Microbiological Analyzer (Sylab, Austria) system, consisted of two incubators allowing four different temperatures simultaneous setting, was used. The strains *L. helveticus* 5, *L. delbrueckii* subsp. *bulgaricus* 202, *Lc. lactis* 4068, and *S. thermophilus* 547 were 10-fold (first dilution), 100-fold (second dilution), 1000-fold (third dilution), 10,000-fold (fourth dilution), 100,000-fold (fifth dilution) diluted in ringer solution (Oxoid Ltd.). Not diluted colture and each dilution were inoculated (2% v/v) into previously sterilized measuring cells filled with 6 ml of SSM.

The impedance measurement was performed at 42°C for *Lactobacillus* and *Streptococcus* strains, and 30°C for *Lactococcus* strains. Subsequently 100 μl of the second dilution was used as inoculum for the analysis of all the 80 strains at their optimum growth temperature.

Moreover, three strains for each species (*L. helveticus* 3, 9, 23; *L. delbrueckii* subsp. *bulgaricus* 260, 265, 3436; *Lc. lactis* 664, 4064, 4067, and *S. thermophilus* 192, 160, 526) were also tested at different temperatures: 32°, 37°, 42°, and 47°C for *Lactobacillus* and *Streptococcus* strains and 20°, 25°, 30°, and 35°C for *Lactococcus* strains For each test, impedance measurement was recorded every 10 min for 80 h. All the analysis were carried out in duplicated. One negative sample, consisting of non-inoculated SSM, was also incubated for each temperature tested.

### Statistical analysis

The means and standard deviations of impedance changes in the medium (M%) data were calculated using SPSS (Version 21.0, SPSS Inc., Chicago, IL, USA) statistical software.

## Results and discussion

### Impedance curve interpretation

Impedance measurement is based on the principle that during microbial growth, metabolic processes produce electrically measurable changes in the growth medium. Milk has itself conductive properties because it is rich in charged compounds, especially minerals and salts (Mucchetti et al., 1994). During lactic acid fermentation, the decrease of lactose and the subsequent increase of lactic acid lower the medium pH and, at the same time, enhance its electrical conductivity as a result of the accumulation of lactate ions during fermentation (Carvalho et al., 2003).

Moreover, acidification of milk changes equilibria of buffer system and solubilizes casein-bound calcium and phosphorous salts. This phenomenon increases conductivity sharply, so there is a positive correlation between increased conductivity and milk acidification due to lactic acid bacteria activity.

This variation of electrical conductivity of milk is proportional to the change in microorganisms number and their metabolic activity and, therefore, microbial growth in milk can be measured (Mucchetti et al., 1994). The BacTrac 4300® system measures two specific impedance values, the *E*-value which is referred to as the impedance change at the electrode surface, and the *M*-Value which is the change in conductivity in medium, SSM in this case (Batrinou et al., 2005).

**Figure 1:**
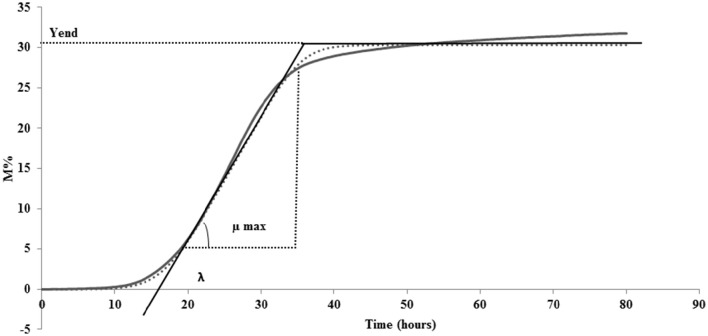
**A generalized impedance growth curve (continues line) and parameters extrapolated from curve by fitting data (dotted line): λ, lag time; μmax, maximum specific M% rate; Yend, maximum value of M%**.

The system enables a separate registration of impedance changes in the SSM (*M*-value) and at the electrode (*E*-value). For the experiments carried out in the present study, the impedance change (*M*-value) of the SSM was used. This value, recorded every 10 min, is revealed as a relative change in the measurement signal and shown as M% percentage in function of time (80 h) in an impedance curve (continues line in Figure 1).

With the aim of translating the metabolic significance of the impedance curve into objective parameters, M% data were fitted to the Modified Gompertz equation (Gibson et al., 1988) using DMfit version 2.1 Excel add-in (http://www.combase.cc/index.php/en/tools). DMfit is part of the system used in-house at the Institute of Food Research to model the time-variation of the logarithm of cell concentration of bacterial batch cultures (www.ifr.ac.uk). Particularly, MS Excel adding DMfit is a free software application for predictive microbiology modeling developed by the Computational Biology Group at Institue of Food Research (Norwik, UK; Perez-Rodriguez and Valero, 2013). Among the primary models available, modified Gompertz equation was used to describe the microbial evolution with time (Swinnen et al., 2004). In this research, the equation was used instead to describe M% in function of time. The fitted data are represented by a sigmoidal curve (shown as dotted line Figure 1) with two inflection points and generate 3 parameters: (i) lag time (λ), (ii) maximum specific M% rate (μmax), and (iii) maximum value of M% (Yend) (Figure 1). The possibility to fit the original data to the Modified Gompertz equation is tied to the necessity that the two curves overlap. All the curves obtained in this study have respected this rule (data not shown).

**Figure 2:**
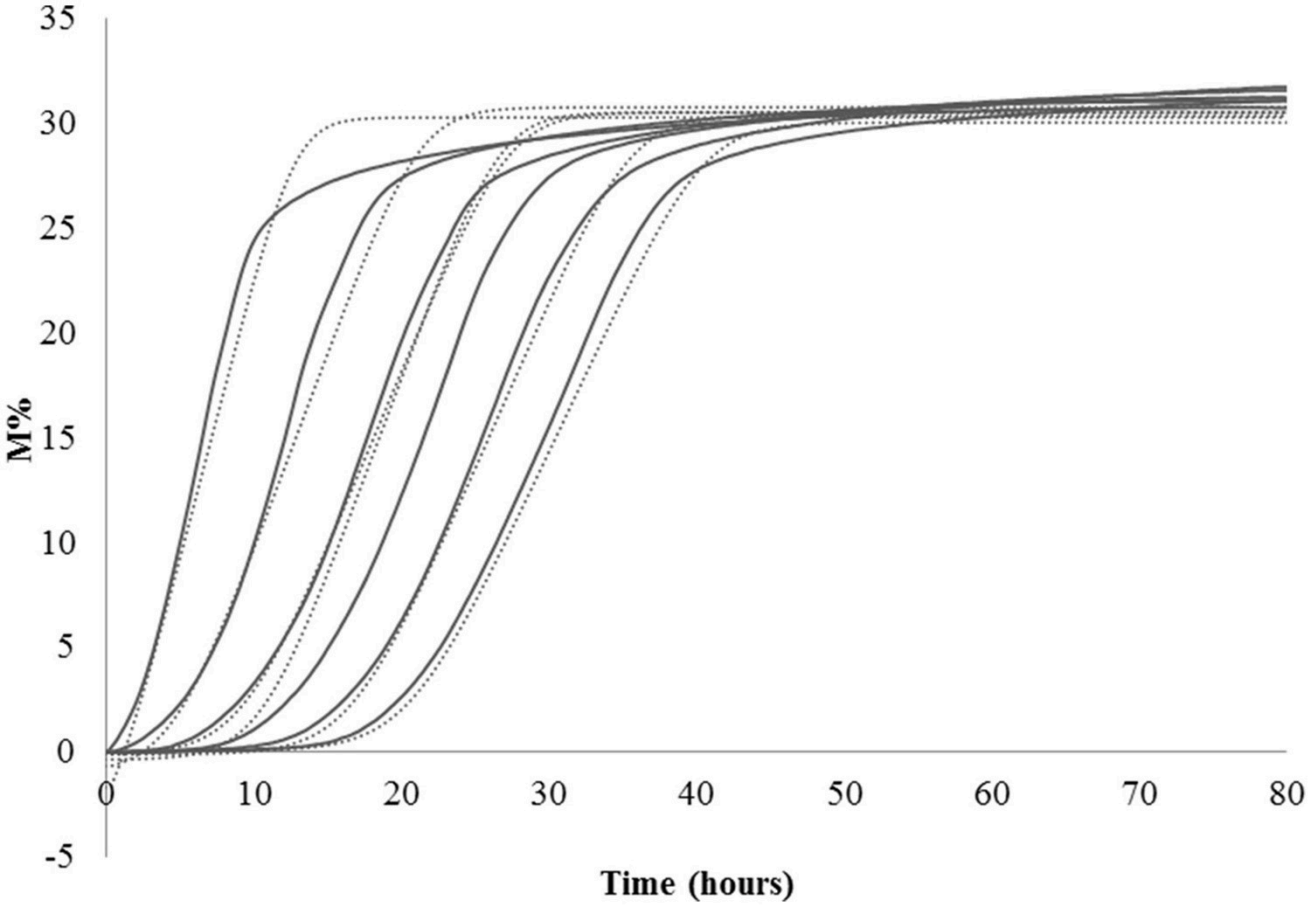
**Impedance curve (continuous line) and impedance curve obtained by fitting data (dotted line) of not diluted colture, first, second, third, fourth, and fifth dilutions**.

Lag phase is an adjustment period during which bacterial cells modify themselves in order to take advantage from the substrate, milk in this case, and initiate exponential growth, so the cells are assumed to be non-replicating (Swinnen et al., 2004). The duration of the Lag phase depends on the strain, temperature and the substrate in which bacteria grow. Many hypotheses have been proposed to describe the formation and duration of the bacterial Lag phase in a growth curve. One of this hypotheses is the individual cell lag time theory (Huang, 2016). Based on this theory, the formation of Lag phase in a bacterial culture is determined by each cell and each cell may leave its lag state individually. Each cell would need to accumulate critical substance before it can grow and start dividing. Once a cell leaves its Lag phase, it enters the exponential phase, starting to grow and divide immediately (Huang, 2016). Based on this concept, lag time (λ) of an impedance curve can be considered as the time that the inoculated cells need to adapt to the condition of the analysis. In the same medium (SSM) at the same temperature (42° and 30°C depending on the species), as expected, for all the species the lower was the inoculum, the greater was the lag time and thus, this parameter is inoculum dependent (Figure 2, Table 2). It has not been possible to register λ value for the inocula of the first and second dilutions of *S. thermophilus* because the time was incompatible with the *minimum* time of registration of the system that needs 1 h to start recording data. During this time, λ values of the first and second dilutions are reached but not recorded.

**Table 2:** **Values of Lag, Rate, and yEnd obtained from the serial dilutions of one strain for species**.

***Inoculum***	***Lactobacillus helveticus 5***			***Lactobacillus delbrueckii* subsp *bulgaricus* 202**			***Lactococcus lactis* 4068**			***Streptococcus thermophilus* 547**		
	**λ (Lag)**	**μmax (M% Rate)**	**yEnd**	**λ (Lag)**	**μmax (M% Rate)**	**yEnd**	**λ (Lag)**	**μmax (M% Rate)**	**yEnd**	**λ (Lag)**	**μmax M% (Rate)**	**yEnd**
Not diluted	0.68	2.64	30.06	1.12	2.33	25.63	1.20	4.25	25.14	nd^‡^	5.27	28.23
First^*^	4.58	1.97	30.55	3.29	2.76	26.98	2.62	4.04	25.27	nd	3.97	28.86
Second^*^	9.01	1.71	30.51	6.78	2.90	26.82	3.97	4.05	26.11	0.33	3.48	29.29
Third^*^	11.45	1.81	30.50	10.48	3.03	27.21	5.53	3.83	26.02	1.59	3.59	29.03
Fourth^*^	16.17	1.59	30.49	14.14	2.97	27.28	7.35	3.77	26.17	2.69	3.65	28.87
Fifth^*^	20.07	1.52	30.13	19.06	2.60	26.86	9.02	3.70	25.87	3.94	3.68	29.42
												
Mean	10.33	1.87	30.38	9.15	2.77	26.80	4.95	3.94	25.76	2.14	3.94	28.95
SD^§^	7.18	0.14	0.22	6.77	0.26	0.60	2.94	0.21	0.45	1.54	0.67	0.42
CV%^†^	69.56	21.77	0.72	74.05	9.51	2.24	59.39	5.25	1.73	72.20	17.00	1.44

* Dilution.

§ Standard Deviation.

† Coefficient of variation.

‡ Not determined.

The second parameter, maximum specific M% rate (μmax) is comparable to the exponential phase and can be used to define LAB fermentation or acidification rate in SSM, which is an important parameter in technological processes, since the greater is the rate, the faster is the acidification. This parameter was inoculum independent as evidenced by the coefficient of variation lower than 10% (Table 2). However, due to the limit of this system that needs 1 h to start recording data, it is better not to use the inocula with highest cell concentrations, such as the undiluted inoculum for *L. helveticus* and *S. thermophilus* because the exponential phase of these cells starts during the BacTrac stabilization. For other devices, which need less time to start recording data, also undiluted inoculum would be used. Considering that the cells divide at a constant rate depending on the composition of the growth medium and the conditions of incubation, the M% rate (μmax) parameter could also be used to determine the time of duplication or generation time. However, as generation time is the time required for microbial cells to double in number (Madigan et al., 2009), to extrapolate the value of generation time from impedance value, a correlation with μmax and number of cells should be carried out.

The third parameter (Yend), is the highest point of the fitted curve, very close to the maximum variation of impedance recorded (Figure 1). This value can be interpreted as the maximum capability of each strain to modify the impedance in SSM and thus depends mainly on its capability to accumulate lactate ions during growth. The capability to accumulate lactate ions can be measured as total amount of lactic acid, as for example, those produced in yogurt, by means of different chemical methods (De Noni et al., 2004). The amount of lactate ions accumulated during growth depends on different aspects, among which, the initial amount of lactose, and acidity tolerance of the strains. In the same medium, with the same amount of initial lactose, Yend can be associated to the acidifying capability and to the resistance of the bacteria to the produced acidity. Of the three considered parameters, Yend is the more independent from the amount of inoculated cells (Table 1).

Considering what has been observed with this first part of the work, if the purpose is to know acidification rate and the amount of produced acid, different inoculum concentrations can be used, getting the same results. However, also considering the minimum time of registration of BacTrac 4300®, the use of a high bacterial concentration, corresponding to the undiluted inoculum or to first dilution, has to be excluded, because it does not allow the visualization of the Lag phase. In this study, we wanted to consider also the λ-value and thus we decide to carry out the analysis with the same inoculum concentration. The highest inoculum that allows the best description of the microbial growth performance in SSM was found to be the second dilution, that has been therefore used for the following determination.

### Impedance analysis of starter LAB at optimal growth temperature

Aiming at evaluating the metabolic significance of the three kinetics parameters λ, μmax, and Yend, 100 μl of the second dilution of 20 strains for each considered LAB species were analyzed in duplicate at their optimal growth temperature, 42°C, for *Lactobacillus* and *Streptococcus*, and 30°C for *Lactococcus*.

**Figure 3:**
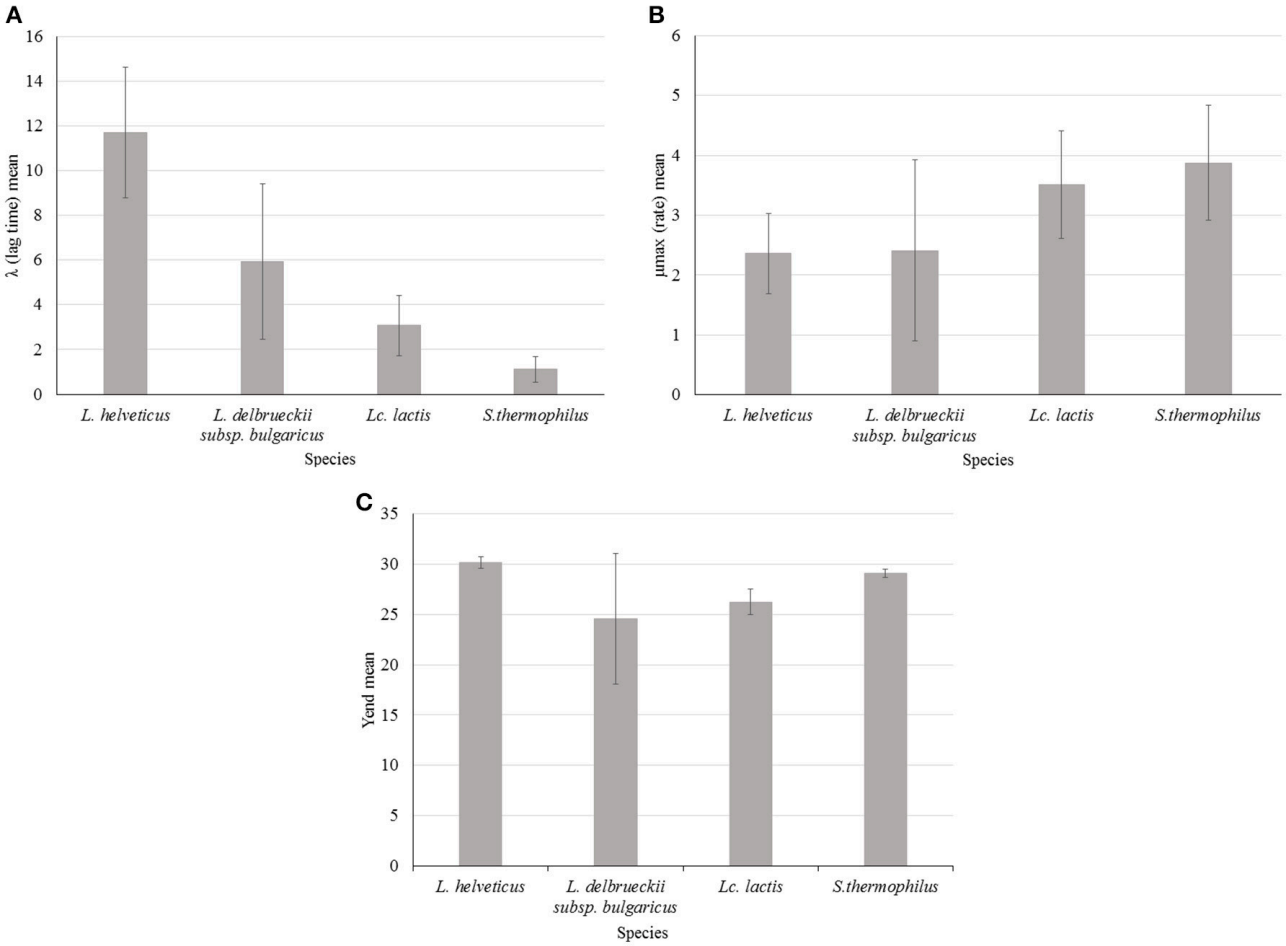
**Impedance analysis of four starter LAB species (***Lactobacillus helveticus***, ***Lactobacillus delbrueckii*** subsp. ***bulgaricus***, ***Lactococcus lactis***, and ***Streptococcus thermophilus***) at optimal growth temperature: (A) λ (lag time) mean value of 20 strains for each species, (B) μmax (rate) mean value of 20 strains for each species, (C) Yend (maximum %M) mean value of 20 strains for each species**. Error bars show standard deviation (SD) for each species.

λ was variable among species and *L. helveticus* showed, on average, the highest values of this parameter. This can be translated into a longer transition period during which the specific growth rate increases to the maximum value characteristic of the culture environment (Swinnen et al., 2004) and thus it can be interpreted as a slower adaptability of the species to the growth condition (Figure 3A). However λ was also highly variable within the species: *L*. *helveticus* and *L. delbrueckii* subsp. *bulgaricus* were the most heterogeneous species, as revealed by standard deviation values (SD) in Figure 3, while *Lc. lactis* and *S. thermophilus* strains showed the lowest and less variable values. Variability of *L. helveticus* in acidifying activity is well known and already measured in different way (Gatti et al., 2003). However, results coming from impedometric analysis and expressed as time of detection, are prone to the variability of the used system. By using the λ value instead, measurements can be made and compared independently from the systems used for the analysis.

Less used in food impedance microbiology but more interesting, if we consider its metabolic significance, is the parameter μmax. Thanks to the approach followed in this work, that is the elaboration of impedometric data by Modified Gompertz equation it is possible to define and compare the different μmax features of the four species. Considering impedance curve of LAB in SSM, high μmax means high acidification rate and *S. thermophilus* and *Lc. lactis* showed higher value in milk respect to *Lactobacillus* species. To one side, this behavior of species is not new (Michel and Martley, 2001; Leroy and Vuyst, 2004) but new and of great interest, is the possibility to easily compare acidification rates among the species and above all among the strains. In this regards, *L. delbrueckii* subsp. *bulgaricus* showed the highest intra-species variability (Figure 3B). This variability could be of great technological interest because among the same species it is possible to choose the strain with higher or lower acidification rate depending on their possible application. For instance, fast acidifying ability can be required for a fermented milk production. On the other hand, lower rate could be desirable for mixed cultures where LAB coexist during changing environmental stimuli and stresses, which can affect their cellular physiology (Arioli et al., 2016).

Variability of maximum acidification rate, calculated measuring pH changing after defined time intervals, for 40 *S. thermophilus* strains, was already observed by Zanatta and Basso (Zanatta and Basso, 1992) using the system Micros (Conegliano, Italy). They found that the maximum acidification rate was the main variable discriminating strains in fast, medium and slow acidifying group (Zanatta and Basso, 1992). More similarly to our approach, a Don Whitley RABIT system (Sherry et al., 2006) was used for a qualitative study of *Salmonella*. μmax for 14 *Salmonella* serovars was determined in less than 7 h, in respect to 24 h needed by conventional method (Sherry et al., 2006).

Different values of Yend, thus different final acidification capability in milk, were found for the four species (Figure 3C). The highest value, with the lowest SD was found, on average, for *L. helveticus*. On the contrary, the lowest value, with the highest SD, was found for *L. delbrueckii* subsp. *bulgaricus*. This means that the first species has the best acidification capability, while the latter has the worst. However, *L. delbrueckii* subsp. *bulgaricus* also showed, as already observed for the others parameters, the more heterogeneous behavior within the species. This can be due to different acid resistance, but it can also be associated with the different capability of the species to metabolize the galactose moiety after lactose uptake. *L. helveticus* is able to ferment the glucose and galactose moieties of lactose (Mollet and Pilloud, 1991), while consumption of galactose by *L. delbrueckii species* depends on the subspecies, and the inability of the subspecies *delbrueckii* and *bulgaricus* to metabolize galactose could be due to the loss of the *galT* gene (Germond et al., 2003). The low average value of Yend and high level of SD measured for *L. delbrueckii* subsp. *bulgaricus*, were due to the presence of at least 6 strains characterized by Yend values lower than 20 (data not shown), possibily linked to the absence of the *galT* gene (Germond et al., 2003). Incapacity to metabolize galactose may be also the reason for lower levels of Yend found for *Lc. lactis* (Figure 3C), confirming that during the metabolism of lactose by *Lc. lactis*, part of the galactose 6P is dephosphorylated and excreted into the growth medium, while the glucose moiety is readily used (Neves et al., 2010). High and homogeneous level of Yend in *S. thermophilus* could be due to galactose positive strains. The existence of galactose negative strains has been reported, but only as a mutation of recent past (de Vin et al., 2005).

Correlation between the two parameters μmax and Yend was not found (data not shown), indicating that fastest strains were not always the greatest acidifying ones. Thus, this method of characterization allow to choose the best strains considering which parameter is the most important for the desired technological application. For example, *L. helveticus* 35 was the best acidifying strain among all studied strains (Yend 31.4) but it was the slowest of its species (data not shown). On the other hand, one of the best acidifying *S. thermophilus* strain, 410 (Yend 29.6), was the faster (rate 5.8) among all studied strains (data not shown).

### Impedance analysis of starter LAB at different growth temperature

Mesophilic bacteria, such as *Lc. lactis*, have an optimum growth temperature of 30°C, while thermophilic species, such as *L. helveticus, L. delbrueckii* subsp. *Bulgaricus*, and *S. thermophilus*, have an optimum growth temperature of 42°C. However, starter LAB employed in dairy fermentations can grow over a wide temperature range varying from 4 to 50°C (Hickey et al., 2015). This aspect is of particular importance because the milk transformations, such as microbial fermentation for the production of fermented milks and acidification of the curd in cheeses production, may involve temperatures quite far from the optimal for bacterial growth.

Considered this, in order to see how starter strains change their performances depending on temperatures, three strains for each species, chosen among the 20 previously evaluated, were tested through impedance analysis under temperatures 5° and 10°C lower and 5°C higher than the optimal for thermophilic species and 5°C lower and 5° and 10°C higher than the optimal for mesophilic species.

**Figure 4:**
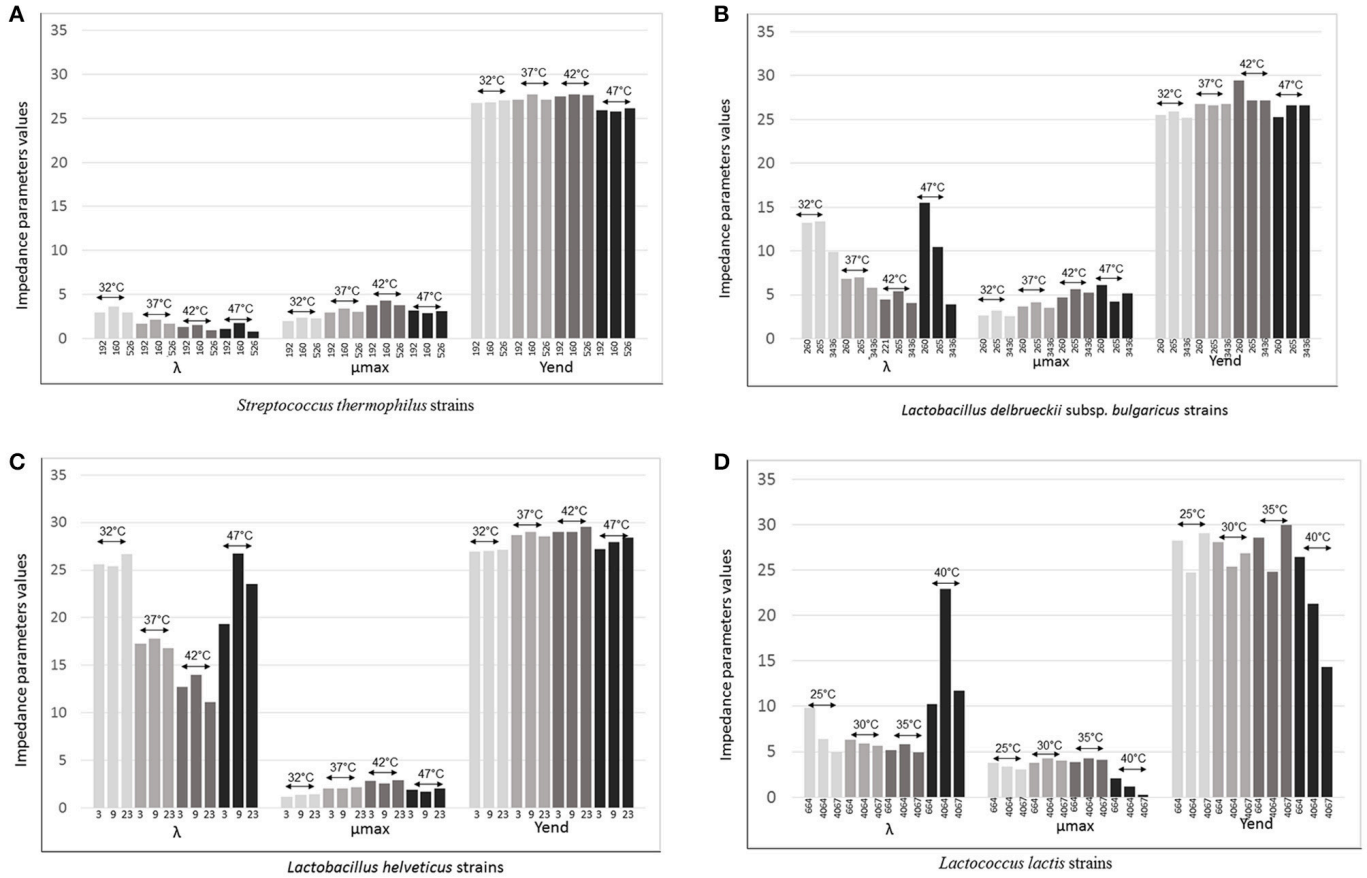
**λ (lag time), μmax (rate) Yend evaluated for (A) Three strains of ***Streptococcus thermophilus*** (192, 160, and 526) evaluated at 32°, 37°, 42°, and 47°C; (B) Three strains of ***Lactobacillus delbrueckii*** subsp. ***bulgaricus*** (260, 265, and 3436) evaluated at 32°, 37°, 42°, and 47°C; (C) Three strains of ***Lactobacillus helveticus*** (3, 9, and 23) evaluated at 32°, 37°, 42°, and 47°C; (D) Three strains of ***Lactococcus lactis*** (664, 4064, and 4067) evaluated at 25°, 30°, 35°, and 40°C**.

Varying temperature, Lag was the parameter that changed greatly. The differences were relevant for *L. delbrueckii* subsp. *bulgaricus, L. helveticus* and *Lc. lactis* and to a lesser extent for *S. thermophilus*, although it was clear that differences were strains dependent (Figures 4A–D). The time of adaptation to the different temperatures was longer when temperature was higher than the optimal both for the *Lactobacillus* species and *Lc. lactis*. On the contrary, it was shorter, when temperatures were lower than optimal. However, the strains *L. helveticus* 3, *L. delbrueckii* subsp. *bulgaricus* 3436, and *Lc. lactis* 664 and 4067 showed to adaptable more easily to the higher temperatures (Figures 4B–D). Of particular interest was the thermal tolerance observed for *L. delbrueckii* subsp. *bulgaricus* 3436 (Figure 4B).

The acidification rate, measured as M% (μmax), was variable for all species in function of the variation of the temperature; at the optimum, it was higher than rates at lower or higher temperatures for all strains (Figures 4A–D). This data is in agreement with the effect of changing temperature on the specific growth rate μ, evaluated by a pH-auxostat study for one strain of *S. thermophilus*, one of *L. delbrueckii* subsp. *bulgaricus* and one of *Lc. lactis* (Adamberg et al., 2003). These authors observed that *S. thermophilus* had the highest specific growth rate at 44°C and a slight decrease at 45°C; 43°C was the temperature at which *L. delbrueckii* subsp. *bulgaricus* reached the maximum rate level, while *Lc. lactis* reached the maximum at 35°C and then, slightly decreased (Adamberg et al., 2003). Interestingly, in the present work, we found exceptions for *L. delbrueckii* subsp. *bulgaricus* 221, that at 47°C acidified faster than at its optimum (42°C; Figure 4B).

All *Lc. lactis* strains, and in particular strain 4067, slowed consistently the rate when incubated at 40°C, while they tolerated the oscillation of 5°, higher and lower than their optimum 30°C (Figure 4D).

The acidifying capacity was not greatly affected by the temperature for the thermophilic species even when incubated at 10°C below the optimum (42°C. Figures 4A–C). In particular, all the three *S. thermophilus* strains have maintained comparable acidification capacity values at each considered temperatures (Figure 4A). Instead, the response of *Lc. lactis* was strictly strain specific at all the temperatures. In particular, *Lc. lactis* 4067 and *Lc. lactis* 4064 showed similar acidification capacity at respectively 40° and 30°C (optimum, Figure 4D).

In an intriguing experiment, it was demonstrated that one *Lc. lactis* strain, mutant TM29, after a long adaptation of 860 generation, was able to grow well up to 39°C due to mutations accumulated, most of which were shown to affect thermal tolerance (Chen et al., 2015). The goal of that research was to demonstrate a simple approach to obtain non-GMO derivatives of *Lc. lactis* that possess properties desirable by the industry, such as thermal robustness and increased rate of acidification. In fact, Chen et al. (2015) report that in the same cheese production, during the curdling process, the temperature is often raised to around 40°C, or even beyond, and in those condition *Lc. lactis* stops growing dramatically, reducing curd acidification. In this perspective, *Lc. lactis* 4067, which by the way, is a wild strain isolated from raw cow milk used for Grana Padano cheese production, could have a great potential industrial interest.

## Conclusion

The impedance microbiology is used since the seventies, but, besides the food control analysis to which it is commonly applied, only few researches had the purpose to study its different potential applications. With this work we wanted to find an objective way to interpret the metabolic significance of impedance curves and propose it as a valid approach to evaluate the potential acidifying performances of starter lactic acid bacteria to employ in milk transformation. The novelty and importance of our findings, obtained by means of BacTrac 4300®, are that they can also be applied to data obtained from other impedometric devices. Moreover, the meaning of Lag, μmax and Yend that we have extrapolated from modified Gompertz equation and discussed for LAB in milk, can be exploited also to other food environment or other bacteria, assuming that they can give a curve and that curve is properly fitted with Gompertz equation Through this study, it was possible to highlight that the LAB species with the highest acidification rate were *S. thermophilus* and *Lc. lactis*, while *L. helveticus* and *S. thermophilus* showed the greatest acidification capacity. Among the 80 studied strains, 20 for each species, the widest heterogeneity was observed within *L. delbrueckii* subsp. *bulgaricus* subspecies. This intraspecific diversity was particularly evident when temperature was far from the optimal. Results obtained for some strains may be of interest for fermented milk and cheese production, particularly for cooked or semi-cooked cheeses.

## Author contributions

EB Substantial contributions to the design of the work; the acquisition, analysis and interpretation of data for the work. VB Substantial contributions to the design of the work and interpretation of data for the work. BB Drafting the work and revising it critically for important intellectual content. EN revising the work critically for important intellectual content and final approval of the version to be published. MG Substantial contributions to the conception and design of the work, interpretation of data for the work, agreement to be accountable for all aspects of the work in ensuring that questions related to the accuracy and integrity of any part of the work are appropriately investigated and resolved.

### Conflict of interest statement

The authors declare that the research was conducted in the absence of any commercial or financial relationships that could be construed as a potential conflict of interest.

## References

[B1] Adamberg K. Kask S. Laht T.-M. Paalme T. (2003). The effect of temperature and pH on the growth of lactic acid bacteria: a pH-auxostat study. Int. J. Food Microbiol. 85, 171–183. 10.1016/S0168-1605(02)00537-8 12810281

[B2] Amorim L. R. Silva J. G. Gibbs P. A. Teixeira P. C. (2009). Application of an impedimetric technique for the detection of lytic infection of *Salmonella* spp. by specific phages. Int. J. Microbiol. 2009:259456. 10.1155/2009/259456 20016810 PMC2789333

[B3] Arioli S. Della Scala G. Remagni M. C. Stuknyte M. Colombo S. Guglielmetti S.. (2016). *Streptococcus thermophilus* urease activity boosts *Lactobacillus delbrueckii* subsp. *bulgaricus* homolactic fermentation. Int. J. Food Microbiol. [Epub ahead of print]. 10.1016/j.ijfoodmicro.2016.01.006 26826763

[B4] Baranyi J. Roberts T. (1995). Mathematics of predictive food microbiology. Int. J. Food Microbiol. 26, 199–218. 10.1016/0168-1605(94)00121-L 7577358

[B5] Batrinou A. M. Katsogiannos E. D. Koustoumpardis E. N. Spiliotis V. K. (2005). Estimation of microbial population of bitter chocolate mix by impedance measurement. Nutrition 29, 260–264.

[B6] Buchanan R. L. Whiting R. C. Damert W. C. (1997). When is simple good enough: a comparison of the Gompertz, Baranyi, and three-phase linear models for fitting bacterial growth curves. Food Microbiol. 14, 313–326. 10.1006/fmic.1997.0125

[B7] Carvalho A. S. Silva J. Ho P. Teixeira P. (2003). Impedimetric method for estimating the residual activity of freeze-dried *Lactobacillus delbrueckii* ssp. *bulgaricus*. Int. Dairy J. 13, 463–468. 10.1016/s0958-6946(03)00049-9

[B8] Chen J. Shen J. Ingvar Hellgren L. Jensen P. Solem C. (2015). Adaptation of *Lactococcus lactis* to high growth temperature leads to a dramatic increase in acidification rate. Sci. Reports 5:14199. 10.1038/srep14199 26388459 PMC4585701

[B9] Chowdhury B. Chakraborty R. Chaudhuri U. (2007). Validity of modified Gompertz and logistic models in predicting cell growth of Pediococcus acidilactici H during the production of bacteriocin pediocin AcH. J. Food Eng. 80, 1171–1175. 10.1016/j.jfoodeng.2006.08.019

[B10] Čurda L. Plocková M. (1995). Impedance measurement of growth of lactic acid bacteria in dairy cultures with honey addition. Int. Dairy J. 5, 727–733. 10.1016/0958-6946(94)00038-Q

[B11] De Noni I. Pellegrino L. Masotti F. (2004). Survey of selected chemical and microbiological characteristics of (plain or sweetened) natural yoghurts from the Italian market. Lait 84, 421–433. 10.1051/lait:2004020

[B12] de Vin D. F. Rådström P. Herman L. Vuyst L. (2005). Molecular and biochemical analysis of the galactose phenotype of dairy *Streptococcus thermophilus* strains reveals four different fermentation profiles. App. Microbiol. Environ. 71, 3659–3667. 10.1128/AEM.71.7.3659-3667.2005 16000774 PMC1168995

[B13] Gatti M. Lazzi C. Rossetti L. Mucchetti G. Neviani E. (2003). Biodiversity in *Lactobacillus helveticus* strains present in natural whey starter used for Parmigiano Reggiano cheese. J. Appl. Microbiol. 95, 463–470. 10.1046/j.1365-2672.2003.01997.x 12911693

[B14] Germond J.-E. Lapierre L. Delley M. Mollet B. Felis G. E. Dellaglio F. (2003). Evolution of the Bacterial Species *Lactobacillus delbrueckii*: a Partial Genomic Study with Reflections on Prokaryotic Species Concept. Mol. Biol. Evol. 20, 93–104. 10.1093/molbev/msg012 12519911

[B15] Gibson A. M. Bratchell N. Roberts T. A. (1988). Predicting microbial growth: growth responses of salmonellae in a laboratory medium as affected by pH, sodium chloride and storage temperature. Int. J. Food Microbiol. 6, 155–168. 10.1016/0168-1605(88)90051-7 3275296

[B16] Gracias K. S. McKillip J. L. (2004). A review of conventional detection and enumeration methods for pathogenic bacteria in food. Can. J. Microbiol. 50, 883–890. 10.1139/w04-080 15644905

[B17] Hickey C. D. Sheehan J. J. Wilkinson M. G. Auty M. A. (2015). Growth and location of bacterial colonies within dairy foods using microscopy techniques: a review. Front. Microbiol. 6:99. 10.3389/fmicb.2015.00099 25741328 PMC4332360

[B18] Huang L. (2016). Simulation and evaluation of different statistical functions for describing lag time distributions of a bacterial growth curve. Microbial. Risk Anal. 1, 47–55. 10.1016/j.mran.2015.08.002

[B19] Kunicka-Styczyńska A. Gibka J. (2010). Antimicrobial activity of undecan-x-ones (x = 2–4). Pol. J. Microbiol. 59, 301–306. 21466049

[B20] Lanzanova M. Mucchetti G. Neviani E. (1993). Analysis of conductance changes as a growth index of lactic acid bacteria in milk. J. Dairy Sci. 76, 20–28. 10.3168/jds.s0022-0302(93)77319-1 8436674

[B21] Leroy F. Vuyst L. (2004). Lactic acid bacteria as functional starter cultures for the food fermentation industry. Trends Food Sci. Tech. 15, 67–78. 10.1016/j.tifs.2003.09.004

[B22] Madigan M. T. Martinko J. M. Dunlap P. V. Clark D. P. (2009). Clark Brock Biology of Microorganisms, 12th Edn. San Francisco, CA: Pearson Benjamin Cummings.

[B23] Marino M. Bersani C. Comi G. (2001). Impedance measurements to study the antimicrobial activity of essential oils from Lamiaceae and Compositae. Int. J. Food Microbiol. 67, 187–195. 10.1016/S0168-1605(01)00447-0 11518428

[B24] Michel V. Martley F. G. (2001). *Streptococcus thermophilus* in Cheddar cheese–production and fate of galactose. J. Dairy Res. 68, 317–325. 10.1017/S0022029901004812 11504394

[B25] Mollet B. Pilloud N. (1991). Galactose utilization in *Lactobacillus helveticus*: isolation and characterization of the galactokinase (galK) and galactose-1-phosphate uridyl transferase (galT) genes. J. Bac. 173, 4464–4473. 2066342 10.1128/jb.173.14.4464-4473.1991 PMC208110

[B26] Mortari A. Adami A. Lorenzelli L. (2015). An unconventional approach to impedance microbiology: detection of culture media conductivity variations due to bacteriophage generated lyses of host bacteria. Biosens. Bioelectron. 67, 615–620. 10.1016/j.bios.2014.09.075 25449877

[B27] Mucchetti G. Gatti M. Neviani E. (1994). Electrical conductivity changes in milk caused by acidification: determining factors. J. Dairy Sci. 77, 940–944. 10.3168/jds.s0022-0302(94)77029-6

[B28] Neves A. R. Pool W. A. Solopova A. Kok J. Santos H. Kuipers O. P. (2010). Towards enhanced galactose utilization by *Lactococcus lactis*. Appl. Environ. Microbiol. 76, 7048–7060. 10.1128/AEM.01195-10 20817811 PMC2976262

[B29] Noble P. A. Dziuba M. Harrison D. J. Albritton W. L. (1999). Factors influencing capacitance-based monitoring of microbial growth. J. Microbiol. Meth. 37, 51–64. 10.1016/S0167-7012(99)00040-8 10395464

[B30] Perez-Rodriguez F. Valero A. (eds.). (2013). Software and data bases: use and application, in Predictive Microbiology in Foods (New York, NY: Springer-Verlag), 75–85.

[B31] Rediers H. Hanssen I. Krause M. Assche A. Vis R. Moloney R. (2012). A whole-chain approach to food safety management and quality assurance of fresh produce, in Progress in Food Preservation, eds Bhat R. Alias A. K. Paliyath G. (Chichester, UK: John Wiley & Sons Ltd.), 429–445.

[B32] Sherry A. E. Patterson M. F. Kilpatrick D. Madden R. H. (2006). Evaluation of the use of conductimetry for the rapid and precise measurement of *Salmonella* spp. growth rates. J. Microbiol. Meth. 67, 86–92. 10.1016/j.mimet.2006.03.004 16616386

[B33] Silva J. L. Wang C. L. Scruggs P. L. Kim S. T. (2003). Impedance microbiology to screen various antimicrobials on whole and fillet channel catfish. J. Rapid Methods Automat. Microbiol. 11, 153–161. 10.1111/j.1745-4581.2003.tb00037.x

[B34] Slongo A. Rosenthal A. Camargo L. Deliza R. Mathias S. Aragão G. de (2009). Modeling the growth of lactic acid bacteria in sliced ham processed by high hydrostatic pressure. Lwt Food Sci. Technol. 42, 303–306. 10.1016/j.lwt.2008.06.010

[B35] Stewart G. N. (1899). The changes produced by the growth of bacteria in the molecular concentration and electrical conductivity of culture media. J. Exp. Med. 4, 235–243. 10.1084/jem.4.2.235 PMC2118043 19866908

[B36] Swinnen I. A. Bernaerts K. Dens E. J. Geeraerd A. H. Van Impe J. F. (2004). Predictive modelling of the microbial lag phase: a review. Int. J. Food Microbiol. 94, 137–159. 10.1016/j.ijfoodmicro.2004.01.006 15193801

[B37] Yang L. Bashir R. (2007). Electrical/electrochemical impedance for rapid detection of foodborne pathogenic bacteria. Biotechnol. Adv. 26, 135–150. 10.1016/j.biotechadv.2007.10.003 18155870

[B38] Zanatta P. Basso A. (1992). A new approach to the characterization of *Streptococcus salivarius* subsp *thermophilus* based on acidification rates. Le Lait 72, 285–295. 10.1051/lait:1992321

